# Oral Lichen Planus: Clinical Features, Etiology, Treatment and Management; A Review of Literature

**DOI:** 10.5681/joddd.2010.002

**Published:** 2010-03-14

**Authors:** Marzieh Boorghani, Narges Gholizadeh, Ali Taghavi Zenouz, Mehdi Vatankhah, Masoumeh Mehdipour

**Affiliations:** ^1^ Assistant Professor, Department of Oral Medicine, Faculty of Dentistry, Tabriz University of Medical Sciences, Tabriz, Iran; ^2^ Lecturer, Department of Oral Medicine, Faculty of Dentistry, Tabriz University of Medical Sciences, Tabriz, Iran

**Keywords:** Oral lichen planus, literature review, clinical features, therapy

## Abstract

Lichen planus is a chronic inflammatory mucocutaneous disease. Mucosal lesions are classified into six clinical forms and there is malignant potential for two forms of OLP; therefore, follow-up should be considered. There are many un-established etiological factors for OLP and some different treatment modalities are based on etiology. The aims of current OLP therapy are to eliminate mucosal erythema and ulceration, alleviate symptoms and reduce the risk of oral cancer. We have used review papers, case reports, cohort studies, and case-and-control studies published from 1985 to 2010 to prepare this review of literature.

## Introduction

Lichen planus is a chronic inflammatory mucocutaneous disease which frequently involves the oral mucosa. In the majority of patients with oral lichen planus (OLP) there is no associated cutaneous lichen planus or lichen planus at other mucosal sites. This may be called “isolated” OLP.^1^ This disease has most often been reported in middle-aged patients 30-60 years of age and is more common in females than in males.^2^ OLP is also seen in children, although it is rare.^3 , 4^

The disease affects 0.5-2% of the population. The clinical history confirms the relationship between OLP and oral cancer, although the degree of the risk involved is controversial. Therefore, OLP should be considered a precancerous lesion, emphasizing the importance of periodic follow-ups in all the patients.^5^

### Clinical Features

OLP was first described clinically by Wilson in 1869 as a chronic mucocutaneous disorder.^6^ Cutaneous lichen planus is recurrent, itchy^7 , 8^ and not contagious.^9^ Concomitant disease involving the scalp, nails, esophageal mucosa, larynx and conjunctivae occurs much less frequently. In many patients, the onset of OLP is insidious, and patients are unaware of their oral condition. Some patients report a roughness of the lining of the mouth, sensitivity of the oral mucosa to hot or spicy foods, painful oral mucosa, red or white patches on the oral mucosa, or oral ulcerations. The clinical history includes phases of remission and exacerbation.^10^

The clinical evaluation of the oral lesions is based on the six clinical forms described by Andreason:^11^ reticular, papular, plaque, atrophic, erosive, and bullous. Mucosal lesions, which are multiple, generally have a symmetrical distribution, particularly on the mucosa of the cheeks, adjacent to molars, and on the mucosa of the tongue, less frequently on the mucosa of the lips (lichenous cheilitis) and on the gums (the atrophic and erosive forms localized on the gums manifest as a desquamative gingivitis), more rarely on the palate and floor of the mouth.^9 , 12^ However, this clinical appearance of desquamative gingivitis is not pathognomonic of erosive OLP and may represent the gingival manifestation of many other diseases such as cicatrical phemphigoid, pemphigus vulgaris, epidermolysis bullosa acquisita, and linear IgA disease.^13 , 14^ The most common type is reticular form with the characteristic feature of slender white lines (Wickham’s striae) radiating from the papules. Patients with reticular lesions are often asymptomatic, but atrophic (erythematous) or erosive (ulcerative) OLP is often associated with a burning sensation and pain.^15^

A greater malignant potential has been recognized for atrophic, erosive form of OLP and the plaques form on the back of the tongue.^5 , 16^ Mignogna et al^17 , 18^ have suggested that regular follow-up of patients with OLP should be performed up to 3 times a year. OLP with dysplasia should be examined more frequently, every 2-3 months. However, patients with asymptomatic, mainly reticular type may be assessed annually. The signs that may be indicative of transformation, such as the extent of symptoms and loss of homogeneity, should be assessed thoroughly at each appointment. When there is evidence of changes in clinical appearance, the follow-up period should be shortened and biopsy should be provided.^17 , 18^

### Etiology

Although the etiology and pathogenesis of OLP are not fully understood, oral lichen planus has been associated with multiple disease processes and agents, such as viral and bacterial infections, autoimmune diseases, medications, vaccinations and dental restorative materials. The association between OLP and chronic liver disease is still controversial. It was first suggested by Mokni et al^19^ in 1991. Carrozzo et al^20^ have demonstrated a strong association between hepatitis C viral infection and OLP. High prevalence rates of HCV infection in patients with OLP have been reported, as high as 62% in Japan^21^ and 27% in southern Italy.^22^ On the other hand, no statistically significant relationship has been found between OLP and hepatitis C in a study which included 30 patients with cutaneous lichen planus, 30 patients with OLP and 30 healthy individuals as the control group in NW Iran.^23^ The results of the study are consistent with the results of a study carried out by Bagan et al^24^ in Spain on 505 patients with hepatitis. However, the association of OLP with HCV infection appears to be dependent on geographical heterogeneity.

Recently, several studies have reported a relationship between Helicobacter pylori and OLP. Moravvej et al^25^ in 2007 found statistically significant differences in H. Pylori infection between patients with lichen planus and a control group. Vainio et al^26^ in 2000 conducted a study on the peptic ulcer and H. pylori in 78 patients with lichen planus and 57 controls and reported differences between the patients with lichen planus and controls; however, the differences were not statically significant. Another study which included 30 patients with cutaneous lichen planus, 30 patients with OLP, and 30 healthy individuals as the control group, could not also establish an etiologic role for H. pylori in lichen planus.^27^

Numerous studies have investigated the prevalence of candidal infection in erosive lichen planus. Recently, a study was carried out on 50 patients with OLP and 35 healthy patients to investigate presence of candida; no significant difference was reported between the two groups.^28^ According to another study, there were no significant differences between the 21 healthy individuals and 21 patients with erosive lichen planus regarding candida presence.^29^ However, candida infection is not currently considered an etiologic factor for OLP.

Exacerbations of OLP have been linked to periods of psychological stress and anxiety.^30 , 31^ In contrast, Humphris & Field^32^ and Macleod^33^ reported no statistically significant association between OLP and anxiety.

Genetic predisposition seems to play a role in OLP pathogenesis as several familial cases have been reported.^34 , 35^ Lowe et al^36^ first reported a significantly high frequency of HLA-A3 in a group of British patients with cutaneous lichen planus. However, Porter et al^37^ reported no significant association with a particular HLA in familial lichen planus.

### Treatment

Generally, no medication is necessary for the benign form of this disease (reticular lichen planus). In the case of severe pain and a burning sensation, high-potency topical corticosteroids remain the most reliably effective treatment modality.^38^ Oral hygiene and corrective dentistry play a major role in the management of OLP and consultation with a dentist or oral medicine specialist is helpful.^39^

### Corticosteroids

Systemic corticosteroids are probably the most effective treatment modality for patients with diffuse erosive OLP or multi-site disease, but the literature on their use is limited to non-randomized clinical trials. Both methylprednisolone and prednisone have been employed for recalcitrant severe erosive OLP.^40^ Systemic prednisone can be used to control the ulcers and erythema in OLP but it is not better than treatment with topical triamcinolone acetonide alone.^41^ Interestingly, topical corticosteroids have been found to be equally or more effective than systemic corticosteroids or the combination of the two. Systemic corticosteroids may be indicated in patients whose condition is unresponsive to topical steroids or in patients with mucocutaneous disease and in high doses (1.5-2 mg/kg/daily), but adverse effects are possible even with short courses.^42 , 43^

Topical corticosteroids are widely used in the treatment of OLP to reduce pain and inflammation. Triamcinolone acetonide is commonly used either in Orabase or as lozenges.^44^ An oral suspension of triamcinolone has also been used with beneficial effects.^45^Hydrocortisone hemisuccinate in aqueous solution seems of little benefit in treating OLP,^46^ whereas betamethasone valerate pellets,^43^ or aerosol,^47^ have been shown to be somewhat effective.

High-potency steroid mouthwashes such as disodium betamethasone phosphate or clobetasol propionate, can be used in extensive OLP but these may be systemically absorbed leading to a pituitary-adrenal axis suppression.^48^ Recently, fluticasone propionate spray has been used effectively in the short-term management of symptomatic OLP, but 10% of the patients did not tolerate such treatment for more than 3 weeks.^49^ Topical corticosteroids in adhesive paste, such as betamethasone valerate, clobetasol, fluocinolone acetonide, fluocinonide, and triamcinolone acetonide, have been widely used.^50 , 51^ The more potent fluorinated steroids include 0.05% fluocinonide and 0.1% fluocinolone acetonide, which have been found to be effective in the treatment of severe OLP that has failed to respond to other medications.^52^ 0.1% fluocinolone acetonide in Orabase has been shown to be more effective than a similar 0.1% triamcinolone acetonide preparation with no serious side effects.^42^ Moreover, the effectiveness of various forms of topical fluocinolone acetonide applications in patients with OLP in a two-year treatment period resulted in complete remission of 77.3%, 21.4%, and 17.0% of patients in the fluocinolone acetonide in Orabase (FAO), fluocinolone acetonide in solution (FAS), and FAS/FAO groups, respectively.^53^ This drug can also be effectively used in the management of lichenoid lesion flare-ups in patients with systemic diseases such as hypertension, heart disease, or diabetes mellitus with no serious side effects.^53^ Clobetasol propionate in aqueous solution, ointment, or Orabase has also been shown to be effective in OLP. Clobetasol can be more effective than fluocinonide in improving lesions,^54^ and the long-term use of clobetasol (6 months) may help control the disease, offering substantial disease-free periods in 65% of the patients after 6 months of follow-up.^54^ Although there are some reports of systemic absorption and adrenal suppression from super-potent topical steroids in the treatment of chronic skin disorders, adrenal suppression has not been reported in long-term oral application of topical corticosteroids such as 0.05% fluocinonide, 0.1% fluocinolone acetonide, and 0.05% clobetasol.^56^ Acute pseudomembranous candidiasis is the only common side effect from topical corticosteroid therapy,^54^ which can be prevented with antifungal (miconazole gel) alone or with chlorhexidine mouthwashes.^53^ In a study on the effect of a mixture of triamcinolone and vitamin A mouthwash with triamcinolone mouthwash alone on the treatment of OLP, either 0.2% triamcinolone acetonide mouthwash with vitamin A or triamcinolone acetonide separately was effective in reducing the number of areas involved, pain, and irritation in oral erosive lichen planus.^57^ According to the results of that study, there were no significant differences in pain severity and burning sensation between the case and control groups. Another study showed that either zinc mouthwash with fluocinolone ointment or fluocinolone ointment separately was effective in reducing the number of areas involved, pain, and irritation of erosive OLP.^58^ Decrease in pain and irritation in both groups was identical and it seems that it can be attributed to the effect of fluocinolone ointment on decreasing pain and irritation, which was used for both groups.^58^ However, decrease in the number of areas involved with zinc mouthwash plus ointment was more than that with ointment alone, which seems to be related to the effect of zinc on healing of impaired epithelium. Intra-lesional injections of hydrocortisone,^59^ dexamethasone,^60^ triamcinolone acetonide,^61^ and methylprednisolone^62^ have been used in the treatment of OLP. However, the injections can be painful, are not invariably effective, and have localized effects, such as mucosal atrophy.^63^ In summary, systemic corticosteroids should be reserved for acute exacerbations and multiple or widespread lesions. Topical treatments can be used with systemic corticosteroids to reduce the systemic side effects or can be used alone. Some studies have suggested that cyclosporine is effective when applied either topically^64^or in the form of a mouthwash,^65 , 66^ but others have reported little benefits^67^ or no significant improvement.^68 , 69^ In OLP patients, systemic absorption is probably low and most studies have not detected cyclosporine in peripheral blood. Although many studies have claimed that cyclosporine is effective, the disadvantages of this medication are bad taste, transient burning sensation on initial application, and high cost.^70^

Comparative study of cyclosporine and triamcinolone acetonide in Orabase in the treatment of OLP has not found any significant differences in remission rates, and another recent comparative study found that clobetasol in adhesive medium is more effective than cyclosporine in the same medium.^71^

Tacrolimus is a potent immunosuppressive agent, inhibiting T-cell activation at 10-100 times lower concentration than cyclosporine. Tacrolimus and pimecrolimus are newer calcineurin inhibitors, with an improved safety profile in comparison with cyclosporine, in the prevention of rejection in organ transplant recipients and graft-versus-host disease (GvHD) in allogeneic hematopoeitic stem-cell transplant recipients. Only a limited number of studies have been published in this regard.^55 , 56^ However, there is a Food and Drug Administration (FDA) “Black Box” warning attached to the use of these agents because of a theoretically increased risk of malignancy (squamous cell carcinoma and lymphoma) in patients using topical tacrolimus/pimecrolimus for cutaneous psoriasis. Recently, the treatment of chronic erosive OLP with low concentrations of tacrolimus has been found to yield a rapid and important palliative effect, but all the patients experienced relapse after a 12-month follow-up period in this study.^72^

### Retinoids

Systemic and topical forms of retinoids have been used in the treatment of OLP. Topical 0.1% vitamin A rapidly eliminated white lesions of OLP but all the cases relapsed 2-5 weeks after discontinuation of treatment.^73 , 74^

###  Ultraviolet Irradiation

Photochemotherapy with 8-methoxypsoralen and long-wave ultraviolet light (PUVA) has been used successfully in the treatment of skin lesions and cutaneous lichen planus.^74 , 75^ It was first used in the treatment of recalcitrant OLP.^76^ Eighty-seven percent of patients treated with ultraviolet-A, without a systemic or topical photosensitizer, improved significantly.^77^ Some studies have indicated that PUVA therapy might also have therapeutic effects.^76^ To avoid PUVA side effects, photosensitization with topical 0.01% trioxsalen can be used for the treatment,^78^ although oral mucosa seems more resistant to phototoxic damage in comparison to skin.^79^ PUVA with 8-methoxypsoralen has various side effects, such as nausea, dizziness, eye symptoms, paraesthesia, and headache.^80^ Photochemotherapy may be useful for severe forms of erosive OLP that do not respond to conventional treatment.^81^ Moreover, one matter of concern is that PUVA therapy has been shown to have oncogenic potential.^82^ Further studies on PUVA therapy for OLP are needed.

###  Laser Therapy

In 2004, Barclay^83^ used 308-nm Excimer laser radiation in OLP patients. This technique was suggested as a viable, palliative treatment option with high patient acceptance in those with symptomatic OLP. In 2005, Soliman et al^84^ used diode laser (980 nm) in the management of OLP as an easy, fast and safe technique. It could be used in an outpatient clinic with local anesthesia. In 2008, van der Hem et al^85^ found good results in the treatment of OLP lesions with CO2 laser evaporation. In this study, there were no problems with wound healing. In every case there was complete epithelialization within 3 weeks. They found out that when there is no further improvement with steroids, CO2 laser evaporation seems to be a good treatment option for oral lichen planus and may even be considered a first choice.
